# Differential Effects of Pravastatin and Simvastatin on the Growth of Tumor Cells from Different Organ Sites

**DOI:** 10.1371/journal.pone.0028813

**Published:** 2011-12-22

**Authors:** David G. Menter, Victoria P. Ramsauer, Sam Harirforoosh, Kanishka Chakraborty, Peiying Yang, Linda Hsi, Robert A. Newman, Koyamangalath Krishnan

**Affiliations:** 1 Department of Cancer Biology, The University of Texas, M.D. Anderson Cancer Center, Houston, Texas, United States of America; 2 Department of Pharmaceutical Sciences, East Tennessee State University, Bill Gatton College of Pharmacy, Johnson City, Tennessee, United States of America; 3 Department of Experimental Therapeutics, The University of Texas, M.D. Anderson Cancer Center, Houston, Texas, United States of America; 4 Department of Cell Biology, The Cleveland Clinic, Cleveland, Ohio, United States of America; 5 Department of Clinical Cancer Prevention. The University of Texas, M.D. Anderson Cancer Center, Houston, Texas, United States of America; 6 Division of Hematology-Oncology, Department of Internal Medicine, East Tennessee State University, Johnson City, Tennessee, United States of America; Bauer Research Foundation, United States of America

## Abstract

3-hydroxy-3-methylglutaryl coenzyme A reductase (HMGCR) inhibitors, commonly known as statins, may possess cancer preventive and therapeutic properties. Statins are effective suppressors of cholesterol synthesis with a well-established risk-benefit ratio in cardiovascular disease prevention. Mechanistically, targeting HMGCR activity primarily influences cholesterol biosynthesis and prenylation of signaling proteins. Pravastatin is a hydrophilic statin that is selectively taken up by a sodium-independent organic anion transporter protein-1B1 (OATP1B1) exclusively expressed in liver. Simvastatin is a hydrophobic statin that enters cells by other mechanisms. Poorly-differentiated and well-differentiated cancer cell lines were selected from various tissues and examined for their response to these two statins. Simvastatin inhibited the growth of most tumor cell lines more effectively than pravastatin in a dose dependent manner. Poorly-differentiated cancer cells were generally more responsive to simvastatin than well-differentiated cancer cells, and the levels of HMGCR expression did not consistently correlate with response to statin treatment. Pravastatin had a significant effect on normal hepatocytes due to facilitated uptake and a lesser effect on prostate PC3 and colon Caco-2 cancer cells since the OATP1B1 mRNA and protein were only found in the normal liver and hepatocytes. The inhibition of cell growth was accompanied by distinct alterations in mitochondrial networks and dramatic changes in cellular morphology related to cofilin regulation and loss of p-caveolin. Both statins, hydrophilic pravastatin and hypdrophobic simvastatin caused redistribution of OATP1B1 and HMGCR to perinuclear sites. In conclusion, the specific chemical properties of different classes of statins dictate mechanistic properties which may be relevant when evaluating biological responses to statins.

## Introduction

Statins may be useful for the prevention and treatment of cancer [1], [2], [3], [4]. Statins were first isolated as fungal metabolites that exhibited potent cholesterol lowering activity through the inhibition of 3-hydroxy-3-methylglutaryl coenzyme A reductase (HMGCR) [5]. These compounds were soon recognized to lower cholesterol through two systemic mechanisms. First, statins reversibly inhibit HMGCR and thus reduce intracellular pools of cholesterol. This results in an increase in low-density lipoprotein (LDL)-receptors on cell surfaces leading to clearance and catabolism of LDL [6]. Certain statins also inhibit hepatic LDL production by preventing the synthesis of the LDL precursor, VLDL [7]. The role of cholesterol in cancer progression remains to be resolved but many tumor cell lines and tissues exhibit higher levels of cholesterol than their normal counterparts [8], [9]. Some reports indicate that hypocholesterolemia occurs in cancer due to increased use of cholesterol by tumors [10] whereas other reports have associated lower tissue cholesterol with malignancy [11].

Statins prevent the rate-limiting conversion of HMG-CoA to mevalonate by HMGCR, which is not only a precursor of cholesterol but is an essential metabolite in the formation of isoprenes. Isoprenes are critical compounds involved in the prenylation of numerous signaling molecules such as small G proteins [12]. Statin mediated inhibition of the prenylation process is reversible by the addition of the various isoprenes such as mevalonate, farnesyl-pyrophosphate, and geranyl-geranyl-pyrophosphate [12]. Prenylation also occurs in many cellular and systemic regulatory pathways that are partly responsible for the pleiotropic effects of statins [13]. Other pleiotropic effects may be independent of prenylation or inhibition of cholesterol production such as cell cycle arrest [14].

Epidemiological studies and meta-analyses of statin use and cancer risk in the general population have provided conflicting results. Some studies of cancers have shown risk reduction associated with statin use [15], [16], [17] while other studies have reported no effect from its use [18], [19], [20] or even an increased risk [21].

The pharmacological features of statins are important in understanding the role of statins in the treatment and prevention of cancer [2], [3], [4]. The lipophilicity of statins and the presence or absence of the transporter molecules on the cell surface can influence the pharmacokinetics and intracellular distribution of statins that affect bioactivity. Pharmacokinetic studies in rats and humans have shown that hydrophilic statins such as pravastatin primarily affect the liver [22], [23]. In humans these liver specific effects rely on a liver specific transporter:organic anion transporter peptide (official gene designation *SLCO1B1*; official protein designation OATP1B1) [24]. This gene is also known as *SLC21A6* and the protein is also known as LST-1, OATP2, OATP-C, or OATP6 [24]. The OATP1B1 transporter is involved in liver specific uptake of pravastatin [25], [26]. It is important to note that genetic polymorphisms may also have a functional impact on OATP/SLCO1B1 [27]. In contrast to hydrophilic statin pharmacokinetic distribution, hydrophobic statins are readily distributed in many tissues [28]. We hypothesized that the hydrophobic-simvastatin is expected to affect a wide variety of tumor cell lines isolated from a variety of organ sites whereas hydrophilic-pravastatin is expected to exhibit liver-specific effects on primary cultures of hepatocytes and liver derived tumor cells. In this study we present the comparative effects of pravastatin or simvastatin on normal hepatocytes as well as in tumor cells isolated from a variety of organ sites.

## Materials and Methods

### Chemicals

Hydrophilic pravastatin and hyrophobic simvastatin were obtained from Calbiochem, San Diego, CA. Calcein acetoxymethyl (CAM) ester, MitoTracker Red CM-H_2_XRos, and 4′-6-diamidino-2-phenylindole (DAPI) were purchased from Molecular Probes-Invitrogen Corporation, Carlsbad, CA. Acetonitrile, ammonium acetate, and Triethylamine, all HPLC grade, were purchased from Fisher (Pittsburgh, PA). Reagent grade formic acid (≥95%), dimethylsulfoxide and additional chemicals were purchased from Sigma Chemical Co. St Louis, MO.

### Cell lines and cell cultures

Pairs of well-differentiated or poorly-differentiated cell lines that originated in multiple tissue sites were obtained from American Tissue Type Culture Collection (ATCC; Manassas, VA) including: colon (Caco-2, HCT-116), pancreatic (Capan-1, MiaPaca), liver (Hep G2, Hep 3B), breast (MCF-7, SKBr-3), prostate (LNCaP, PC-3), bladder (U-9, U-14), skin (SCC-M7, SCC-P9) and lung cancer (Calu-3, Calu-6) cell lines. Tumor cell lines from different epithelial origins were grown in tissue culture according to ATCC instructions. Normal human hepatocytes were purchased from Cambrex BioScience (Walkersville, MD). Primary cell cultures were maintained in defined hepatocyte growth medium according to distributor's instructions. Cell cultures were routinely tested for mycoplasma by RNA/DNA hybridization (Gen-Probe, San Diego, CA), and treated if needed with BM-Cyclin from Roche (Indianapolis, IN).

### Analysis of cell viability, apoptosis and mitochondrial distribution by fluorescence microscopy

Apoptosis and nuclear morphology, DNA dye uptake, and cellular staining were assessed by fluorescence microscopy. Cells were plated in 96-well plates and treated with 0, 0.1, 1, 5, 10, and 20 µM pravastatin or simvastatin for 6 h, 24 h, 48 h, and 72 h. Treatments were performed in 0.5% fetal bovine calf serum in the appropriate medium. Cell viability was determined at each time point by staining with vital dye CAM ester (2 µM) in phenol red free DMEM for 15 minutes at 37°C. Cells were simultaneously incubated with MitoTracker Red CM-H_2_XRos (1 µM), and DAPI (1 ng/ml) Molecular Probes). Nuclear morphology, DNA dye uptake, and cellular staining were assessed by fluorescence using an Olympus IX-70 inverted microscope. Image acquisition was achieved using a Quantix charged coupled device camera and IP Labs software (Scanalytics, Inc., Fairfax, VA) on a Macintosh computer (Apple Computer Corporation Cupertino CA).

### Immunofluorescence analysis

Normal hepatocytes were established as monolayers on laminin-coated coverslips to perform immunofluorescence studies. Cells were treated with 10 µM pravastatin or simvastatin for 72 hr. Cells were fixed in 1% paraformaldehyde and processed for immunofluorescence studies as described before [29]. Briefly, cell monolayers grown on cover slips were immunolabeled with anti-OATP1B1, anti-p-cofilin (Santa Cruz Biotechnology, Santa Cruz, CA), anti-p-caveolin (BD Biosciences Pharmingen, San Jose, CA), anti-HMGCR (Upstate Biotechnology, Lake Placid, NY) and Alexa488 labeled secondary antibody; Molecular Probes, Eugene, OR followed by counterstaining to detect DNA with DAPI and actin Alexa 594-phalloidin (Molecular Probes, Eugene, OR). The images were collected and analyzed as described above.

### Cell proliferation assay

Cells were seeded in 96-well plates and treated with 0, 0.1, 1, 5, 10, and 20 µM pravastatin or simvastatin for 6 h, 24 h, 48 h, and 72 h. Proliferation was measured by treating cells with 40 µL of a phosphate buffered saline (PBS) solution containing 2.5 mg/mL 3-(4,5-dimethylthiazol-2-yl)-2,5-diphenyltetrazolium bromide (MTT) followed by removal of medium and solubilization of formazan crystals with 100 µL dimethylsulfoxide. Plates were quantified by reading absorbance at a wavelength of 540 nm on a 96-well Spectramax M5-multiwell plate reader (Molecular Devices, Sunnyvale, CA).

### Reverse Transcriptase-Polymerase Chain Reaction

The RNA STAT-60 reagent (Tel-Test, Inc., Friendswood, TX) was used to extract the total RNA, which was treated with DNase I prior to use in a reverse transcriptase-polymerase chain reaction (RT-PCR) analysis. One microgram of RNA was reverse transcribed with mouse mammary tumor virus RT (Life Technologies, Inc., Rockville, MD). OATP1B1 565 bp sequences were amplified by primer set OATP2-565F 5′-ACTGATTCTCGATGGGTTGG-3′ (forward) and OATP-565R 5′-GTCCGGCAACTGATTTGTTT-3′ (reverse). The 565 bp primer sets and additional primer sets were designed and verified using Oligo 6.7 from Molecular Biology Insights (Cascade, CO). Primer pairs (5′-CAGCTCTGGAGAACTGCTG-3′; 5′-GTGTACTCAGTCTCCACAGA-3′) were used in RT-PCR analysis to detect GAPDH mRNA.

### Western Blot Analysis

Whole cell lysates were prepared as previously described [29]. Briefly, 50 µg of protein was loaded in each lane and run on a NuPAGE Novex precast mini-gels (Invitrogen, Carlsbad, CA) and transferred onto a nitrocellulose membrane (Schleicher & Schuell Bioscience, Inc., Keene, NH). After blocking with 3% fatty acid free-bovine serum albumin, the blots were exposed to antibodies against OATP1B1 or HMGCoA-reductase (Upstate Lake Placid, NY), followed by the appropriate secondary antibody (Pierce Chemical Co., Rockford, IL). The signals were detected by using an enhanced chemiluminescence system (Pierce).

### Uptake of Statins by high performance chromatography and Tandem mass spectrometry (LC/MS/MS)

Statins were analyzed using a modification of previously published methods [30]. After 6 hr of treatment with pravastatin and simvastatin, hepatocyte and PC-3 cells were washed with cold PBS and scraped free in the presence of a lysis buffer containing 20 mM MOPS, 2 mM EGTA, 5 mM EDTA, 30 mM NaF, 40 mM β-glycerophosphate, 20 mM sodium pyruvate, 0.5% Triton X-100, and 1 mM sodium orthovanadate with protease inhibitor cocktail (Sigma). Cell lysates were then sonicated on ice for 3 minutes and transferred to glass tube (13×100 mm). Additional 150 µl of PBS were added to the samples followed by addition of an aliquot of 20 µl of 1 N citric acid. Statins were extracted with 2 ml of ethyl acetate three times. The upper organic phases were pooled and evaporated to dryness under a stream of nitrogen at room temperature. Samples were then reconstituted with 100 µl of 80% of 20 mM ammonium acetate in 0.02% formic acid and 20% acetonitrile before being analyzed by LC/MS/MS. Protein levels were quantified via the DC protein assay (BioRad, Inc., Hercules, CA).

Pravastatin and simvastatin were detected using a Quatro Ultima mass spectrometer (Waters Corp., Milford, MA) equipped with an Agilent 1100 binary LC inlet. Statins were separated using a Hypersil GOLD C_18_ 3 µm column (50×2.1 mm; Thermo Electron, Bellefonte, PA). The mobile phase consisted of 20 mM ammonium acetate, 4 mM triethylamine, and 0.02% formic acid in DI water (solution A) and acetonitrile (solution B). The flow rate was set at 300 µl/minute with a column temperature of 50°C. The gradient for separating the two statins was as follows: 0–2 min. at 90% A, 2 to 2.1 min. linear increase to 100% B, 2.1 to 5 min at 100% B, 5 to 5.1 min. back to 90% A, 5.1 to 9 min. 90% A. The sample injection volume was 25 µl with samples being kept at 18°C in a refrigerated autosampler.

Pravastatin and simvastatin were detected using electrospray negative ionization mode, cone voltage was 60 V, cone gas flow 70 L/hour, and desolvation gas flow at 700 L/hour. Desolvation temperature was 350°C, and the source temperature was 125°C. Fragmentation of all compounds was performed using argon as the collision gas at a cell pressure of 2.1×10^−3^ torr with collision energy setting of 18. Statins were detected using multiple-reaction monitoring of the transition ions 423.40>303.2 and 435.4>319.2 for pravastatin and simvastatin, respectively. Statin concentrations were normalized to the protein content in the samples.

### Data Treatment and Statistical Analysis

Two-way ANOVAs, followed by Tukey's test, were used with the factors cell type and treatment (SAS 9.2; SAS Institute, Cary, NC). Statistical significance was set at p<0.05.

## Results

### Hydrophobic-simvastatin inhibits tumor cell growth more effectively than hydrophilic-pravastatin

**Figure 1:**
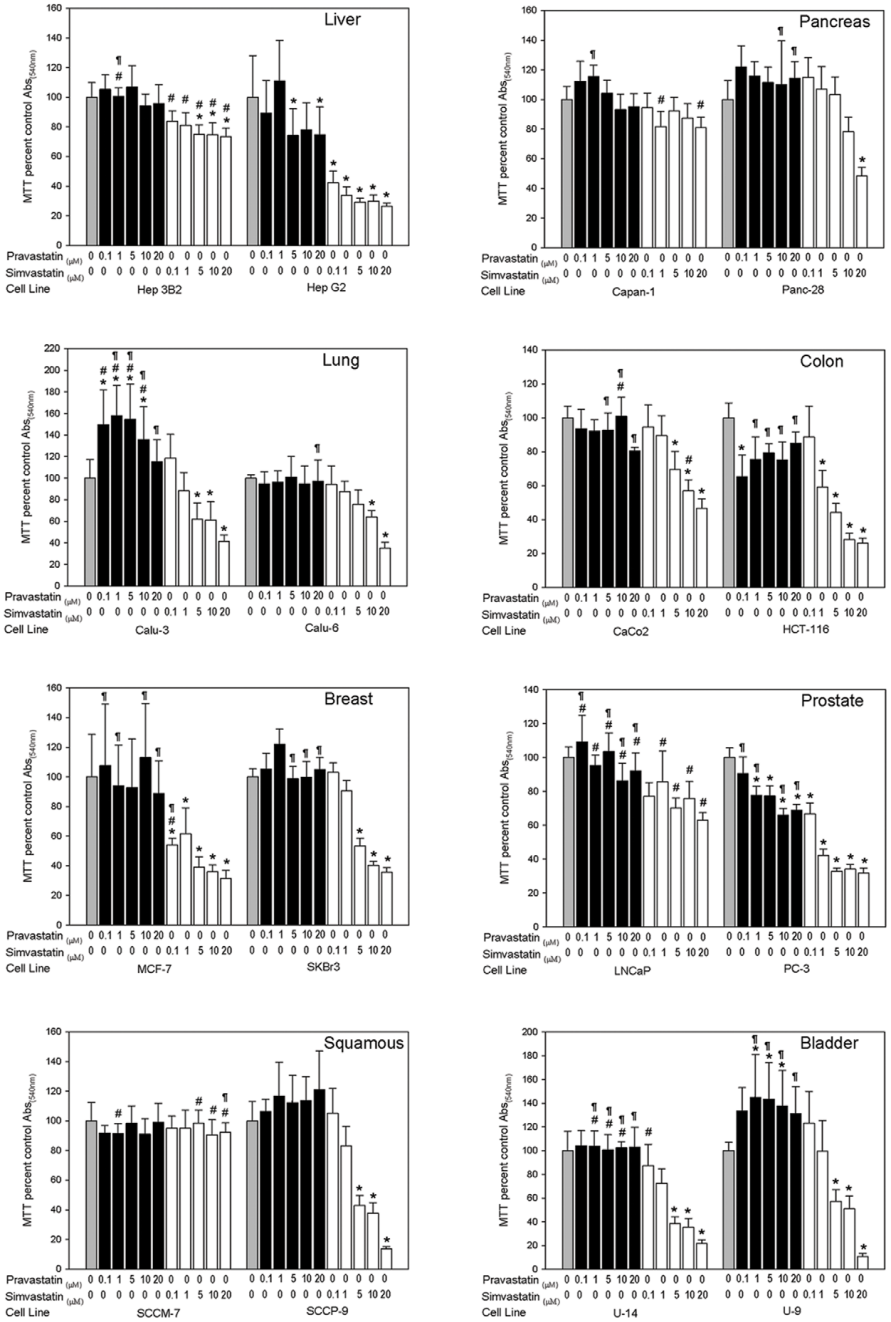
Comparison of the effects of hydrophilic-pravastatin and hydrophobic-simvastatin on normal hepatocytes and cancer cells. The effects of pravastatin and simvastatin on liver hepatocytes and tumor cells was determined by MTT assay and are represented as a percent of the control absorbance at a wavelength of 540 nm. All data were performed at 72 h except for Panc 28 which was responsive at 24 h. Data shown are from representative experiments (n = 8; except colon, n = 4). Values are expressed as mean+SD. * p<0.05, significant difference between control and simvastatin or pravastatin groups. ¶ p<0.05, significant difference between simvastatin and pravastatin groups. # p<0.05, significant difference between well differentiated and poorly differentiated cell types.

Based on MTT assays, simvastatin effects were more pronounced than pravastatin on the growth of all the tumor cell lines examined except for squamous cancer cell line SCCM7 and pancreatic cancer cell line Capan-1(Figure 1). The cell lines tested included malignant colon, pancreatic, prostate, bladder, skin and lung. All experiments were conducted for 72 hours except for the most sensitive cell line Panc 28, which was responsive at 24 hours. Simvastatin exhibited a dose and time dependent inhibition of cancer cell growth, while pravastatin showed minimal or no effect on all of the cancer cell lines studied. Unexpectedly, the typical response to simvastatin was greater in poorly-differentiated cells when compared to the well-differentiated cells as shown in figure 1.

### Simvastatin causes extensive cell shape change and mitochondrial redistribution within hours in highly responsive cancer cells

**Figure 2:**
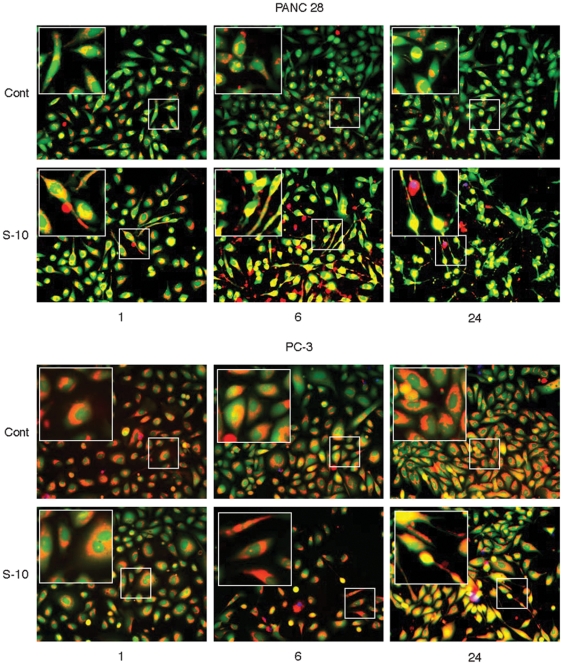
Simvastatin induced shape change and mitochondrial redistribution in cancer cells at early time points. PC-3 prostate cells and Panc 28 pancreatic cells were treated with 10 uM simvastatin and stained with calcein AM (green), MitoTracker CM-H2XRos, a reduced, non-fluorescent dye that fluoresces (red) upon oxidation, and DAPI, a staining dye that emits (blue) when bound to DNA. These images of PC-3 and Panc 28 were acquired via fluorescence microscopy and illustrate that changes in cell shape occurred within 6 hours becoming more extensive by 24 h. These shape changes were accompanied by a significant redistribution of mitochondria.

Image analysis of cells stained with three fluorescent markers was carried out at 1, 6 and 24 h after treatment with 10 µM simvastatin. Calcein AM is a cell permeable vital dye that is cleaved by non-specific esterases to become impermeable to the intact cell membrane and emits a bright green fluorescence. MitoTracker Red CM-H_2_XRos is a reduced, non-fluorescent dye that emits bright red fluorescence upon oxidation. This dye also stains mitochondria in live cells and its accumulation is dependent upon membrane potential. DAPI readily enters cells when plasma membrane integrity is lost and specifically labels nuclei. Examination of two highly sensitive cell lines, PC-3 and Panc 28, illustrate how extensively these cell lines change shape in response to 10 µM simvastatin over a 24 h time period. These changes in cell shape are accompanied by a significant redistribution of mitochondria that initially migrate into cellular processes and coalesce into perinuclear deposits. Panc 28 cells displayed the highest response by extending cellular processes as early as 1 h, a behavior that became more extensive at 6 h and 24 h (Figure 2A). A significant number of Panc28 cells were dead by 24 h. PC-3 cells exhibited similar but less extensive shape changes and behavior over the same time frame (Figure 2B).

### Simvastatin but not pravastatin induced morphological changes and death of tumor cells at 48 h and 72 h

**Figure 3:**
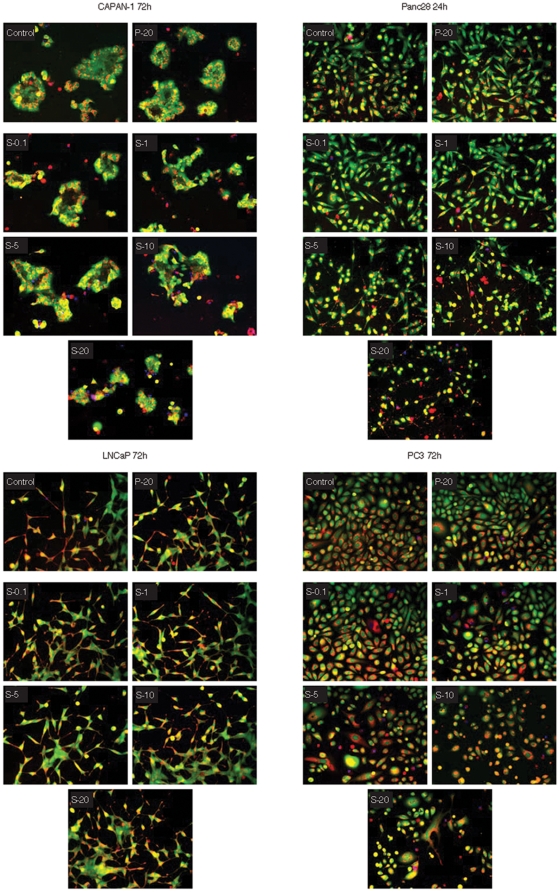
Simvastatin but not pravastatin dose-dependently induces morphological changes and apoptotic behavior in responsive tumor cells. Image analysis of cells stained with three fluorescent markers calcein AM (green), DAPI (blue), and MitoTracker Red CM-H2XRos (red), was performed as described in the methods section. Tumor cells were examined at 72 h except in the case of Panc 28 which were processed at 24 h.

Pravastatin treatment at 20 µM for longer time periods of 48 h (data not shown) and 72 h (Figure 3) had no effect on any tumor cells. In contrast, simvastatin at 48 h and 72 h caused cells to retract their processes and lose plasma membrane integrity (Figure 3). Increased membrane permeability led to leakage of CAM in conjunction with the influx of DAPI. DAPI labeling of DNA illustrated nuclear condensation indicative of apoptosis. Shrinkage of the central cell body around the nuclei and apoptotic body formation is seen at the plasma membrane.

### The expression of HMGCR does not consistently correspond to statin responsiveness

**Figure 4:**
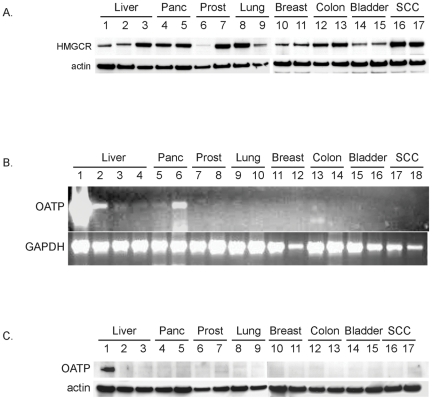
3-hydroxy-3-methylglutaryl coenzyme A reductase (HMGCR) expression varies between cells but the organic anion transporter (OATP) is expressed exclusively in normal liver. A) HMGCR was examined by Western analysis for its expression as follows: liver (normal hepatocytes, lane 1; HepG2, lane 2; Hep3B, lane 3), pancreas (Capan 1, lane 4; Mia Paca, lane 5), prostate (LnCap, lane 6; PC-3, lane 7), lung (Calu3, lane 8; Calu6, lane 9), breast (MCF-7, lane 10; SkBr3, lane 11), colon (Cacao 2, lane 12; HCT116, lane 13), bladder (U9, lane 14; U14, lane 15), or squamous cell carcinoma (SCCP9, lane 16; SCCM7, lane 17). These data illustrate that HMGCR expression does not correspond to drug response. B) Total RNA isolated from human liver tissue (lane L) or cells and analyzed for expression of a 565 bp amplimers from OATP. The numeric sequence of PCR samples is the same as described for protein in A. GAPDH primers were used on the same series of mRNA samples to determine the quality and loading consistency of PCR products. Only whole liver (L) and hepatocytes (1) expressed multiple OATP amplimers by PCR. *Note:* PCR amplimers observed in pancreatic samples (lane 6) were not present when other HMGCR primer sets were used. C) Western analysis revealed OATP protein only in the human liver hepatocytes in lane 1. Tumor cell total protein was examined in the same sequence as in A and did not reveal any OATP protein.

Since HMGCR activity is regulated by statins, total protein isolated from cells was examined by Western blot analysis for HMGCR expression (Figure 4A). The majority of cells derived from a given tissue expressed HMGCR at relatively similar levels regardless of differentiation status, such as pancreatic carcinoma cells (Capan 1, lane 4; MiaPaca, lane 5), breast (MCF-7, lane 10; SkBr3, lane 11), colon (Caco 2, lane 12; HCT116, lane 13), bladder (U9, lane 14; U14, lane 15), or squamous cell carcinoma (SCC-P9, lane 16; SCC-M7, lane 17). In other instances, there were differences in HMGCR expression between well-differentiated and poorly-differentiated cells, as observed in liver hepatocarcinoma cells (HepG2, lane 2; and Hep3B, lane 3), and lung (Calu3, lane 8; Calu6, lane 9). In the prostate, PC-3 cells (lane 7) expressed high levels of HMGCR and responded to statins, whereas LnCAP cells (lane 6) with only traces of HMGCR remained unresponsive to statins. These data suggest that the expression levels of HMGCR in tumor cells do not always correspond to responsiveness to statin treatment and that other factors may be involved.

### Normal hepatocytes but not tumor cells express OATP1B1

Liver-specific organic anion transporter protein OATP1B1 was examined in normal liver hepatocytes and all of the other tumor cell lines since it was reported to mediate liver specific uptake of pravastatin [25]. Normal human liver and normal hepatocytes were the only samples to specifically express OATP1B1 mRNA (Figure 4B) or OATP1B1 protein (Figure 4C). All other tumor cells did not express OATP1B1 mRNA (Figure 4B) or OATP1B1 protein at detectable levels (Figure 4C). Although PCR amplimers were sometimes observed in pancreatic samples (lane 6), further analysis of total RNA by RT-PCR using primers to other regions of OAT-P1B1 revealed specific products only in the human liver tissue and liver hepatocytes.

### Normal hepatocytes incorporate hydrophilic-pravastatin more effectively than tumor cells

**Figure 5:**
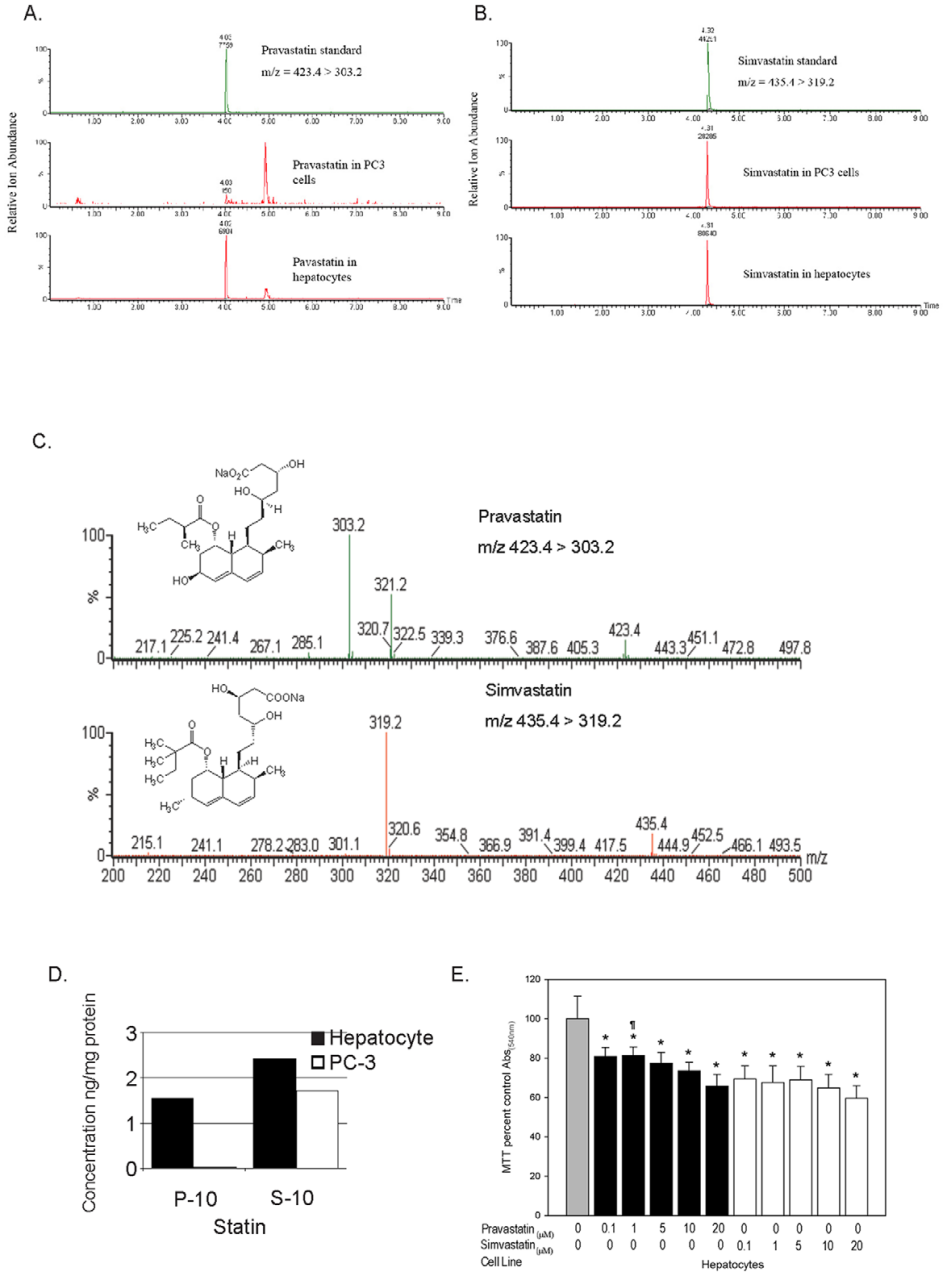
Incorporation and growth response of normal hepatocytes or tumor cells to hydrophilic-pravastatin or hydrophobic-simvastatin. A & B) Total ion chromatography methods were developed to attain critical separation profiles showing pravastatin and simvastatin. C) Mass spectroscopy was performed by using deuterated standards as internal controls for separation to distinguish between statins. Statins were detected by using electrospray-negative ionization and monitoring by magnetic resonance microscopy. Fragmentation of the statins were performed using argon as the collision gas at a collision cell pressure of 2.1×10^−3^ torr. D) LS/MS/MS determination of statins. Monolayers of normal hepatocytes or prostate cancer cells (PC-3) were placed in fresh serum-free medium before the addition of 10 µM pravastatin or simvastatin. Cell culture medium and cells were collected 6 h after treatment. The statins were subjected to solid-phase extraction and analyzed for the presence of pravastatin or simvastatin by LC/MS/MS analysis. Data represent two determinations run in duplicate. E) Effects of pravastain and simvastatin on OATP expressing human hepatocytes were determined by MTT assay and are represented as a percent of the control absorbance at a wavelength of 540 nm. Data shown are from representative experiments (n = 8). These data illustrate that both pravastatin and simvastatin suppressed the growth of hepatocytes to nearly the same extent. Values are expressed as mean+SD. * p<0.05, significant difference between control and simvastatin or pravastatin groups. ¶ p<0.05, significant difference between simvastatin and pravastatin groups.

LC/MS/MS analytical methods were developed to achieve critical separation and identification of pravastatin and simvastatin (Figure 5A and 5B). Mass spectroscopy analysis was done using standards as internal controls to establish separation parameters for pravastatin and simvastatin (Figure 5B). Analysis of statin uptake by normal human hepatocytes or selected tumor cells was performed to determine if OATP1B1 expression correlated with drug incorporation. Cells were seeded on 10 cm dishes and treated overnight with 10 µM pravastatin or 10 µM simvastatin. Cells were lysed and subjected to extraction and determination of pravastatin and simvastatin by LC/MS/MS analysis (Figure 5B and 5C). Human hepatocytes incorporated significantly higher (86 fold) levels of pravastatin (1.55 ng/mg) compared to PC-3 prostate cancer cells (0.018 ng/mg) on a per milligram basis. The difference in hydrophobic-simvastatin uptake between hepatocytes (2.42 ng/ml) compared to PC-3 tumor cells (1.40 ng/ml) was only 1.72 times higher (Figure 5D).

### Normal hepatocytes respond to both pravastatin and simvastatin

Both pravastatin and simvastatin suppressed the growth of OATP1B1 expressing hepatocytes to nearly the same extent (Figure 5E). More simvastatin was incorporated into hepatocytes than pravastatin, which correlated with their effects on suppression. In contrast, tumor cells, which lack OATP1B1 failed to incorporate and did not respond to pravastatin but were growth suppressed by simvastatin (Figure 1). These data illustrate that uptake of pravastatin by OATP1B1 expressing normal hepatocytes correlated with changes in growth and behavioral responses that were absent from tumor cells, which lack OATP1B1 expression.

### Pravastatin as well as simvastatin causes relocalization of OATP1B1 to the perinuclear space in hepatocytes

**Figure 6:**
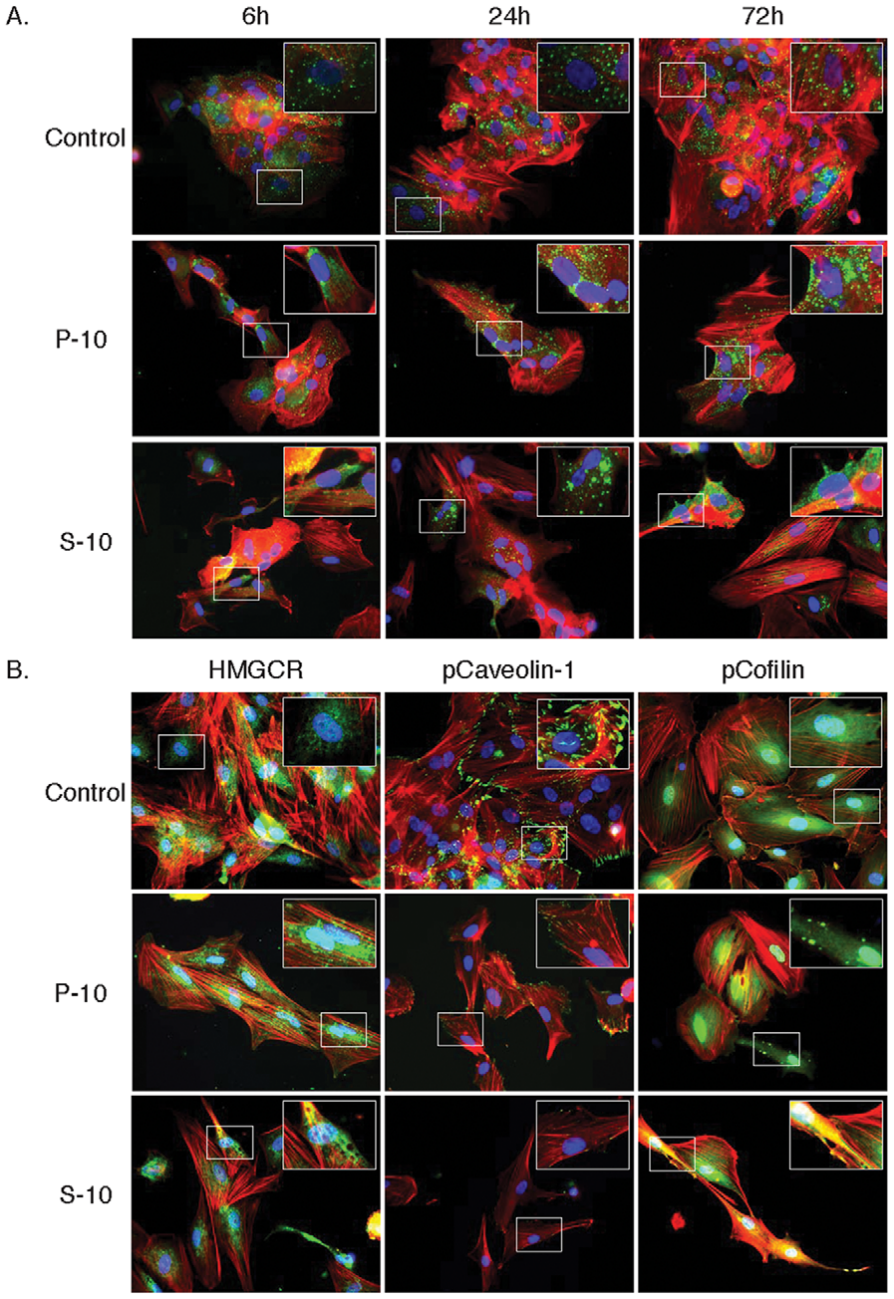
OATP and HMGCR redistribution, loss of p-caveolin and clustering of p-cofilin in statin treated cells. A) OATP immunofluorescent labeling (green) is observed diffusely distributed over the surface of untreated hepatocytes but becomes more perinuclear in cells treated with hydrophilic-pravastatin or lipophilic-simvastatin. Cells were counterstained for actin using alexa-594-phalloidin (red) and nuclear DNA using DAPI (blue). Actin redistribution occurs in conjunction with cellular elongation. B) HMGCR immunofluorescent labeling (left column, green) was diffusely distributed within untreated hepatocytes but became more perinuclear in cells treated with hydrophilic-pravastatin or lipophilic-simvastatin. Cells were counterstained for actin using alexa-594-phalloidin (red) and nuclear DNA using DAPI (blue). P-Tyr14-caveolin (green) expression was lost following statin treatment (middle column). P-Ser3-cofilin (green) formed clusters following statin treatment (right column).

Prior to treatment with statins, OATP1B1 was diffusely distributed in hepatocytes (Figure 6A, top row). After treatment with pravastatin (Figure 6A, middle row) or simvastatin (Figure 6A, bottom row) OATP1B1 progressively became distributed in the perinuclear space of the cell cytoplasm.

### Pravastatin and simvastatin cause altered distribution of HMGCR in hepatocytes

HMGCR distribution was diffuse in control hepatocytes (Figure 6B, left column). After treatment with pravastatin (Figure 6B, middle row) or simvastatin (Figure 6B, bottom row) for 72 h, HMGCR distribution condensed within the perinuclear space in areas resembling the endoplasmic reticulum.

### Pravastatin as well as simvastatin disrupt caveoli in hepatocytes

Inhibition of cholesterol synthesis following statin treatment was expected to disrupt caveoli. Immunofluorescent detection of pY-14-caveolin was examined to determine if statin treatment was capable of disrupting caveoli (Figure 6B, middle column). The observed distribution of pY-14-caveolin in control hepatocytes illustrated the membrane clusters of phospho-protein that were extensively associated with actin filaments. Treatment of hepatocytes with pravastatin (Figure 6B, middle row) or simvastatin (Figure 6B, bottom row) for 72 h illustrated the loss of pY-14-caveolin that coincided with the cytoplasmic process extension and subsequent change in cell shape.

### Pravastatin and simvastatin cause clustering of phospho-cofilin in hepatocytes

Since prenylation of proteins such as G-proteins, Rac and Rho, are affected by statin treatment [12], we examined the downstream target cofilin to determine the effects of statins on its phosphorylation state (Figure 6B, right column). Cofilin phosphorylation on serine 3 was very diffuse in control cells but formed extensive clusters after treatment of hepatocytes with pravastatin (Figure 6B, middle row) or simvastatin (Figure 6B, bottom row). These data suggest that changes in cell morphology that occur during responses to statins may involve cofilin-mediated mechanisms.

## Discussion

We tested the hypothesis that cell type specific uptake of statins influences responsiveness to the drug. We expected hydrophobic-simvastatin to inhibit a wide variety of tumor cell lines compared to hydrophilic-pravastatin which was expected to exhibit liver-specific effects. Hydrophilic-pravastatin was ineffective at inhibiting the growth or altering the biologic behavior of any tumor cell line. Normal hepatocytes were the only cells examined that expressed OATP1B1, a liver specific transporter molecule capable of taking up hydrophilic-pravastatin. Uptake of hydrophilic-pravastatin by normal hepatocytes initiated changes in cellular morphology and caused growth arrest. Neither HepG2 nor Hep3B hepatocarcinoma cells expressed OATP1B1. Thus, neither was able to respond to hydrophilic-pravastatin. In contrast, hydrophobic-simvastatin inhibited the growth of a wide variety of tumor cells in a dose-dependent manner. Unexpectedly, hydrophobic-simvastatin inhibited the growth of cells generally thought to be poorly-differentiated more effectively than those thought to be well-differentiated. Growth inhibition by hydrophobic-simvastatin was accompanied by extensive morphological changes and the redistribution of mitochondria at early time points of 1 h, 6 h and 24 h. Treatments of poorly-differentiated tumor cells for longer time periods 48 h and 72 h caused extensive cell death that was less apparent in the well-differentiated tumor cells.

The expression of OATP1B1 occurs exclusively in normal liver on the basolateral (sinusoidal) plasma membrane of hepatocytes and has 12 transmembrane domains [31]. The role OATP1B1 plays in liver function is to drive the hepatic clearance of albumin-bound amphipathic organic compounds [32]. Studies on tissue samples revealed decreased levels of OATP1B1 in hepatocellular carcinoma tumor samples when compared to normal liver [33], [34]. We observed the absence of detectable levels of OATP1B1 expression by western blot in HepG2 and Hep3B hepatocarcinoma cells, which may explain the lack of response to pravastatin (Figures 1 and 4). Other reports have shown that OATP1B1 expression was lower in HepG2 cell lines compared to normal hepatocytes [33], [35]; however we are unaware of similar observations in Hep3B cells. Other studies on HepG2 cells have shown that pravastatin had less of an effect on cholesterol synthesis than either simvastatin or lovastatin [36]. These results are consistent with our observations of differential inhibition of cell growth in HepG2 and Hep3B hepatocarcinoma cells. Only one other report that we are aware of has examined OATP1B1 in a limited number of tumor cells by RT-PCR [37]. These findings are in line with our observations on the absence of detectable expression levels of OATP1B1 in the tumor cell lines tested.

The OATP1B1 protein exhibits a broad range of transport substrates that includes bile salts, bilirubin, bromosulphophthalein, steroid conjugates, the thyroid hormones T4 and T3, eicosanoids, cyclic peptides, and toxins such as microcystin and phalloidin [38]. It also transports many drugs including benzylpenicillin, methotrexate, rifampicin, and most notably pravastatin. Pravastatin is hydrophilic due to the presence of the hydroxyl group attached to its decalin ring, whereas simvastatin has a methyl group substituted in this position making it hydrophobic. The hydrophilic nature of pravastatin accounts for its minimal penetration into the intracellular space of nonhepatic tissues and does not accumulate in plasma due to first-pass hepatic elimination even with repeated administration [39]. Pharmacokinetic studies have shown that pravastatin is preferentially taken up by liver tissue [23]. The uptake of pravastatin was observed to be OATP1B1 mediated in normal human hepatocytes but not in HepG2 cells [36]. Compared to hydrophilic-pravastatin, hydrophobic-simvastatin is taken up in many tissues in a less selective fashion. Koga *et al* [23] showed pravastatin inhibited sterol synthesis by 90% in the liver and ileum of rodents but less than 14% in kidney, spleen, adrenal, testis, prostate and brain, whereas lovastatin and simvastatin inhibited this process in all tissues. Our observations using LC/MS/MS analysis were consistent with these findings (Figure 5 A–D). Hepatocytes actively incorporated both pravastatin and simvastatin by 6 h and responded to both treatments to the same extent by 72 h (Figure 5E). In comparison, PC-3 prostate cancer cells incorporated far less pravastatin by 6 h (Figure 5 A–D) and were less growth inhibited by 72 h in tissue culture (Figure 1). This was confirmed by LC/MS/MS analysis where the presence of the transporter in hepatocytes accounted for an 86-fold increase in pravastatin uptake as compared to PC3 cells. The response of PC-3 cells by 72 h is likely indicative of pravastatin incorporation by passive diffusion since the drug remained in tissue culture over the full 72 h treatment period and therefore was not subjected to systemic first-pass hepatic elimination. In fact, PC-3 and CaCo2 cells were the only lines to exhibit any type of noteworthy response to pravastatin treatment (fig. 1).

We observed a two-phased response in tumor cells to hydrophobic-simvastatin treatment. The first phase involved a dramatic change in cell morphology within the first 6 h to 24 h. In most cases the death of cells was not very extensive during the early phase (figure 2). The second phase of the response occurred between 24 h to 72 h, which involved the loss of plasma membrane integrity. The early phases of growth arrest appear to involve isoprenylation of small G. proteins. The prenylation process seems to have effects on the biology of individual cells prior to cholesterol depletion and is reversible by the addition of the various isoprenes such as mevalonate, farnesyl-pyrophosphate, and geranyl-geranyl-pyrophosphate [12].

The second phase of cellular response to hydrophobic-simvastatin involves cholesterol depletion. At the cellular level, this can reduce the content of lipid rafts [40] as well as the expression of caveolin-1 [41], [42]. This protein is involved in the formation and regulation of caveolae which are membranous pits that play a role in cellular transport, signaling and cancer [43]. Caveolin can be phosphorylated on tyrosine 14 by Src to form dimers that initiate interactions with the actin cytoskeleton and maintain the structural organization of caveoli [44], [45]. Inhibition of cholesterol synthesis ultimately leads to the induction of apoptosis [46]. We observed downregulation of pY14-caveolin following treatment with either pravastatin (Figure 6B, middle row) or simvastatin (Figure 6B, bottom row) indicative of loss of actin interactions. This coincided with the changes in membrane structure associated with process extension and the change in cell shape, a likely effect of statins on small G proteins. Downstream of G-protein activation lies cofilin, an actin-binding protein involved in the regulation of cell shape and motility [47]. Previous studies have shown that cofilin phosphorylation at serine3 leads to loss of actin binding and severing activities. Cell shape changes and motility require cofilin activation at the leading edge and inactivation in other areas. Taken together, these data implicate the involvement of cofilin-mediated mechanisms in the morphological changes that occur during response to statins (Figure 6B). These data also suggest that the eventual loss of cholesterol in combination with prolonged loss of isoprenylation of signaling factors contribute to morphological changes that ultimately lead to cell death (Figure 6B). These observations corroborate and present additional information to the mechanism of action of statins presented by Gruruswamy et al., in colon cancer cells [42].

Systemic effects of statins are more complex. When the serum cholesterol decreases, a compensatory increase in tissue mevalonate occurs in the extra-hepatic tissue [48]. Duncan *et al.,* have shown that mevalonate promotes the growth of tumors derived from human breast cancer cells in mice [49]. Since simvastatin uptake into extra-hepatic tissue occurs through passive diffusion, such deleterious effects are not expected to occur with the lipophilic statins like simvastatin since mevalonate is depleted in extra-hepatic tissue as well. This corroborates with the observation from epidemiological studies of a lack of adverse effects in terms of cancer risk with the lipophilic statins [50], [51]. A decrease risk of cancer has been noted with the use of the lipophilic statins, simvastatin [52] [17] and lovastatin [51]. On the other hand, the uptake of pravastatin is dependent on the presence of a OATP1B1 [39]. This transporter is not present in extra-hepatic tissue and therefore pravastatin is able to inhibit HMGCR in liver and ileum only where the transporter is present [23]. This leads to an increase in mevalonate synthesis in extra-hepatic tissue and promotes tumor growth of neoplastic cells [23]. This finding may explain the increased incidence of cancers noted in some epidemiological studies with pravastatin [53], [54]. Hence epidemiological studies have to identify the class of statins being analyzed for cancer risk reduction before determining their efficacy. In an editorial in the *Journal of Clinical Oncology*, Kim points out that the association of statin use and cancer risk based on currently published epidemiologic studies and meta-analyses of cancer risks in clinical trials, is inconclusive at its best and that there are no effects at its worst [18]. The recently published epidemiological studies by Jacobs *et. al.,* [20] and Dale *et. al.,* [19], as well as others [17], [55], [56] do not adequately consider this important pharmacological distinction in their analysis. Jacobs *et. al.,* (28) showed no positive effect of statins on cancer risk in a large population of 132,000 men and women in the Nutrition Cohort of the ACS Prevention Study II (CPS-II). The authors point out that a small reduction in risk or an effect of a specific type or dose of statin cannot be ruled out. This study is in marked contrast to the Molecular Epidemiology of Colorectal Cancer (MECC) study in Northern Israel [15], which showed a 47% relative risk reduction in the incidence of colon cancer irrespective of the class of statin used. As outlined by McLaughlin [57] in an editorial, accompanying the Dale *et. al.,* [19] study, future epidemiological studies should draw information not only from large cohorts through self-report but these data should be verified through pharmacy data-bases and population-based cancer registries to obtain details of drug dose, regimes, class of drugs and cancer type and stage. Furthermore, recent reports suggest that associations between statin use and the occurrence of cancer remain inconclusive [58]. The general consensus seems to be that cancer incidence should continue to be monitored among statin users and that longer-latency effects remain possible [58].

*In vitro* experiments with statins have shown significant effects on cell growth and proliferation. We have shown significant differential effects of simvastatin and pravastatin on cell growth and apoptosis in a variety of malignant cancer cell lines. The lipophilic class of statins have profound effects on cell growth and apoptosis in a variety of malignant cell lines. Furthermore, these effects appear to be more profound in poorly-differentiated cancer cell lines. We believe that these differential effects are due to the inability of hydrophilic pravastatin to achieve adequate intracellular concentrations since all malignant cell lines examined lack the expression of the transporter protein required to transport pravastatin into the cell. Regardless of the fact that the statin concentration range used in these experiments is above those observed in clinical pharmacodynamic studies [59], the findings presented here have implications for interpretation and conduct of epidemiological, prevention and treatment studies on the use of statins in cancer. The type and dose of statins used would be important to analyze in epidemiological studies. Based on our findings, we believe that the data from epidemiological studies reported so far do not provide us sufficient ground to eliminate statins as potential chemopreventive or therapeutic agents. Even if the lipophilic statins are not considered to be useful as preventive agents, we have shown the potent cytotoxic properties of the lipophilic class of statins. This may suggest a role for lipophilic statins in the treatment of malignancies in conjunction with other cytotoxics or biological agents. Further studies of the lipophilic class of statins in animal models are necessary to test their efficacy and potential role in cancer therapy or prevention.
